# The use of biparametric magnetic resonance imaging in active surveillance of prostate cancer

**DOI:** 10.1002/bco2.70281

**Published:** 2026-09-18

**Authors:** Carlos A. Rivera López, Michelle I. Higgins, Yuezhou Jing, Mark N. Alshak, Joseph G. Cheaib, Mufaddal Mamawala, Patricia Landis, Mohammad E. Allaf, Katarzyna J. Macura, Christian P. Pavlovich

**Affiliations:** ^1^ The James Buchanan Brady Urological Institute and Department of Urology Johns Hopkins School of Medicine Baltimore Maryland USA; ^2^ Department of Radiology Johns Hopkins School of Medicine Baltimore Maryland USA

**Keywords:** active surveillance (AS), biopsy, biparametric magnetic resonance imaging (bpMRI), diffusion‐weighted imaging (DWI), dynamic contrast enhanced (DCE), gadolinium, Gleason grade group (GG), multiparametric magnetic resonance imaging (mpMRI), prostate cancer, Prostate Imaging‐Reporting and Data System (PI‐RADS)

## Abstract

**Objectives:**

This study aims to evaluate the performance of biparametric magnetic resonance imaging (bpMRI) compared to multiparametric MRI (mpMRI) for active surveillance (AS) in prostate cancer (PCa), focusing on lesion detection, PI‐RADS classification changes and grade reclassification (GR) rates.

**Methods:**

We retrospectively reviewed our institutional AS database for men who underwent mpMRI followed by bpMRI. Lesion counts and PI‐RADS classification were compared. Second, a subgroup of men who underwent mpMRI, biopsy, bpMRI and then repeat biopsy (‘bpMRI biopsy group’) was matched to a control group who underwent mpMRI, biopsy, another mpMRI and then repeat biopsy (‘mpMRI biopsy group’) based on age, PSA density, year of diagnosis and number of positive cores. GR rates at systematic and targeted biopsy of all PI‐RADS 3–5 lesions were compared, as were PI‐RADS changes.

**Results:**

A total of 129 men with a median of 29 months between mpMRI and bpMRI were included. mpMRI identified 77 PI‐RADS 3–5 lesions versus 114 on subsequent bpMRI (0.60 vs. 0.88 lesions/patient, *p* = 0.01); however, lesion distribution was similar, with rates of PI‐RADS 3, 4 and 5 lesions of 53%, 40% and 6% on mpMRI and 53%, 37% and 11% on subsequent bpMRI (*p* = 0.87, 0.29 and 0.23, respectively). In the matched biopsy subgroups, when comparing men who had two surveillance mpMRIs with those who had a surveillance mpMRI followed by a surveillance bpMRI, lesion stability was not significantly different across PI‐RADS categories. GR at last biopsy occurred in 29% of the bpMRI biopsy group versus in 25% of the mpMRI biopsy group (*p* = 0.7).

**Conclusions:**

bpMRI appears safe to incorporate into AS over the short term, as it was noninferior to mpMRI with similar GR rates and lesion stability.

## 1 INTRODUCTION

Active surveillance (AS) is the preferred management for patients with low‐risk prostate cancer and select cases of favourable intermediate‐risk disease. Landmark trials such as PIVOT and ProtecT have demonstrated the safety of surveillance approaches, which delay or avoid radical treatment without compromising outcomes.^1^, ^2^

Historically, the assignment of risk categories and patient monitoring for prostate cancer relied on digital rectal examinations, transrectal prostate biopsies and prostate specific antigen (PSA) monitoring. Now, multiparametric magnetic resonance imaging (mpMRI), which utilizes anatomical T2‐weighted imaging, functional diffusion‐weighted imaging (DWI) and dynamic contrast enhanced (DCE) imaging, is well established for diagnosing and monitoring prostate cancer during AS.^3^, ^4^ mpMRI improves the detection of clinically significant disease and guides targeted biopsies.^5^, ^6^ Practices have shifted to using ultrasound MRI fusion biopsies, and the high accuracy of mpMRI makes it effective both for guiding biopsies and for long‐term patient monitoring.^7^ For all these reasons, mpMRI is commonly used in AS—it helps select which patients may benefit from a for‐cause biopsy when there is adverse MRI change, thereby potentially reducing the number of recurrent biopsies, and makes biopsies more accurate.

Patients on AS typically undergo serial MRIs, which can lead to repeated exposure to intravenous (IV) gadolinium. Recent studies have demonstrated gadolinium accumulation in the human brain following repeated administrations of gadolinium‐based contrast agents, regardless of an intact blood‐brain barrier or normal renal function.^8^ For this and other reasons, there have been concerns within the AS community due to the inherent nature of typical follow‐up protocols, which involve serial mpMRI scans.^9^ Biparametric MRI (bpMRI), which omits the DCE phase, has gained interest for its shorter scan time, lack of IV contrast and comparable diagnostic performance.^10^

In the Prostate Imaging‐Reporting and Data System (PI‐RADS), DCE does not contribute to the overall assessment when the imaging finding has a low (PI‐RADS 1–2) or high (PI‐RADS 4–5) likelihood of representing clinically significant cancer. However, when the DWI score is indeterminate (PI‐RADS 3) in the peripheral zone, a positive DCE increases the score to PI‐RADS 4 to reflect high likelihood that the finding corresponds to a clinically significant cancer.^11^ Therefore, in AS practices where all PI‐RADS 3 or greater lesions are biopsied, the DCE phase may have a limited role.

While bpMRI has shown promise in biopsy‐naïve patients, few studies have evaluated its role in AS.^12^, ^13^, ^14^ We have started incorporating bpMRI into our AS program, generally in men who have at least two prior mpMRIs. This study aims to provide insights into the effectiveness of bpMRI compared to mpMRI in monitoring prostate cancer progression and guiding biopsy decisions in our patients.

## 2 MATERIAL AND METHODS

### 2.1 Study design

**FIGURE 1:**
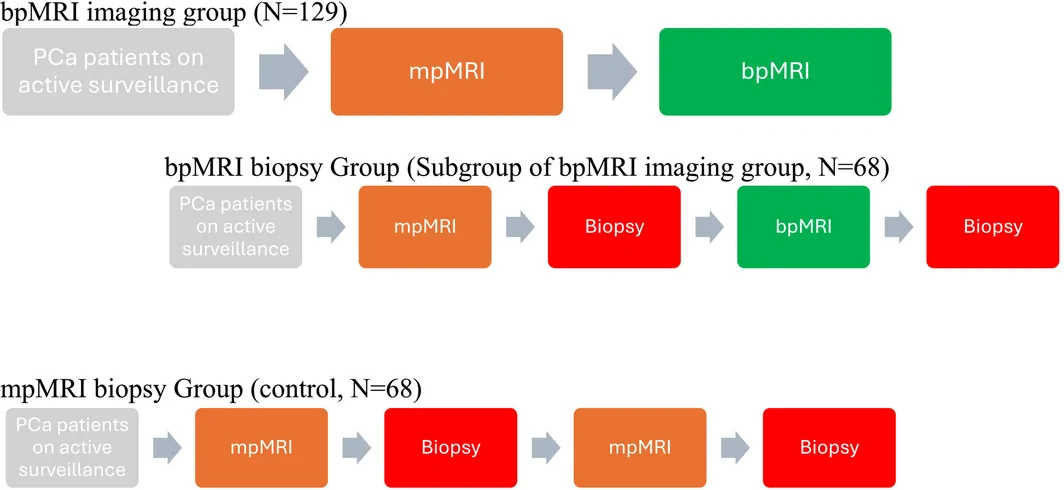
Timeline of the three groups defined in the study.

A retrospective analysis was conducted of low risk and favourable intermediate risk prostate cancer patients on AS who underwent at least one mpMRI followed by a bpMRI between 2016 and 2024. Patient demographic information, average time between MRIs and number of positive PI‐RADS scores^3^, ^4^, ^5^ for lesions were collected. We called this 129‐patient group the bpMRI imaging group (Figure 1).

We aimed to evaluate the pathological outcomes of prostate biopsies in relation to the type of MRI used. To achieve this, we identified a subgroup of patients within the bpMRI imaging group who underwent an mpMRI followed by a biopsy, then a bpMRI and subsequently a second biopsy, in that specific sequence. These patients all had a prior positive prostate biopsy and were in AS. We called this 68‐patient subgroup the bpMRI biopsy group. Note that this group was significantly smaller than the bpMRI imaging group because the introduction of bpMRI into our program was relatively recent, which did not allow time for some patients to get to their next surveillance biopsy. Additionally, a separate group was selected from our institutional AS database consisting of patients who had an mpMRI followed by a biopsy, another mpMRI and a second biopsy, in that same sequence. This group was meticulously matched with the bpMRI biopsy group based on age, PSA density at the time of diagnostic biopsy, the date of the first positive biopsy and the number of positive cores. We called this 68‐patient group the mpMRI biopsy or control group (Figure 1).

We compared several outcomes between groups. These included the rates of grade group reclassification (GR) to any higher Gleason grade group (GG) at second biopsy, extreme grade reclassification (the subset of GR events to GG3 or higher on the second biopsy) and PI‐RADS lesion stability. By analysing these parameters, we aimed to determine whether the type of MRI (mpMRI vs. bpMRI) had a significant impact on the pathological outcomes and overall diagnostic accuracy in patients on AS for prostate cancer. With the mpMRI biopsy group, we compared the evolution of MRI lesions from the first to the second MRI. Since the mpMRI biopsy group acted as our control group, we compared the matched group with the bpMRI biopsy group. We evaluated all lesions on the first MRI and then described their evolution on the subsequent scan (mpMRI in the mpMRI biopsy group and bpMRI in the bpMRI biopsy group). In PI‐RADS 3 and 4 lesions, we determined if the lesions were downgraded, stable or upgraded. In the PI‐RADS 5 lesions, we determined if the lesions were stable or downgraded. We also did an analysis of lesion evolution of PI‐RADS 4 and 5 lesions as a group and PI‐RADS 3, 4 and 5 lesions as a group.

A per lesion analysis was performed on all PI‐RADS 3–5 lesions identified in each group on the second MRI. Lesions that did not undergo documented targeted biopsy were excluded. GR was defined relative to the patient's initial grade group (GG1 or GG2) and was based solely on the pathology of the targeted biopsy core. GR was defined as GG1 to GG2 or higher, or GG2 to GG3 or higher. GR observed only in systematic cores was not considered an event for this analysis.

All MRI scans were interpreted by experienced radiologists as part of routine clinical care in a high‐volume prostate MRI centre. Fusion prostate biopsies were performed by expert urologic surgeons, all as systematic (12–16 cores) + targeted biopsies (3–4/target) when a target was present, and all pathology specimens were reviewed by the institutional Genitourinary Pathology team.

### 2.2 Statistical analysis

Fisher's exact test was used to compare GR and extreme GR rates between the study group (bpMRI biopsy group) and the control group (mpMRI biopsy group) at second biopsy. Additionally, Fisher's exact test was used to assess the differences in the number of PI‐RADS 3–5 lesions between the first (mpMRI) and second (bpMRI) MRI scans within the bpMRI imaging group. Chi‐square testing was used to compare the evolution of PI‐RADS 3–5 lesions between the bpMRI and mpMRI biopsy groups. Noninferiority testing was done using the Farrington‐Manning test comparing PI‐RADS 3, 4 and 5 outcomes between mpMRI biopsy group and bpMRI biopsy group. Finally, T‐testing was used to determine whether the baseline characteristics (such as mean age, mean number of positive cores on the first and second biopsy, mean PSA density at diagnostic biopsy and the median year of initial diagnosis) were comparable between the study and control groups. We defined statistical significance as a *p*‐value of <0.05.

## 3 RESULTS

The study included 129 men on AS at a median age of 70 years (IQR: 65–74 years) and with a median interval of 29 months (24–38 months) between mpMRI and bpMRI. We compared the mean number of lesions per patient in each imaging study and analysed the PI‐RADS classification evolution between mpMRI and follow up bpMRI. A total of 77 PI‐RADS 3–5 lesions were identified on mpMRI, and 114 lesions on subsequent bpMRI, resulting in an average of 0.60 lesions on mpMRI and 0.88 lesions per patient on bpMRI, respectively (*p* = 0.01). While the number of lesions increased over the 29‐month median interval between MRIs, the distribution of lesions was similar between mpMRI and bpMRI, with PI‐RADS 3, 4 and 5 lesions comprising 53%, 40% and 6% of lesions on mpMRI and 53%, 37% and 11% on bpMRI, respectively. There were no statistically significant differences in PI‐RADS score distributions between the two MRI modalities (Table 1).

**TABLE 1:** PI‐RADS lesion evolution in patients that had an initial mpMRI followed by a bpMRI.

bpMRI imaging group (*N* = 129)			
	Initial mpMRI	Follow‐up bpMRI	*p*‐value
Total number of PI‐RADS lesions^3^, ^4^, ^5^	77	114	
Mean number of lesions per patient	0.6	0.88	0.01
Total number of PI‐RADS 3 lesions	41(53%)	60 (53%)	0.867
Total number of PI‐RADS 4 lesions	31(40%)	42 (37%)	0.288
Total number of PI‐RADS 5 lesions	5 (6%)	12 (11%)	0.225
Median interval between MRIs (months)		29	

To evaluate lesion evolution from the first MRI to the follow‐up MRI, we compared results from the bpMRI biopsy group to those from the mpMRI biopsy (control) group. The groups were well matched by relevant prostate cancer parameters. There were no significant differences between the bpMRI and mpMRI biopsy groups in the number of patients with no lesions on the first MRI (33 vs. 28, *p* = 0.41), nor in the development of new lesions on the second MRI among those patients (20 vs. 11, *p* = 0.12). No significant differences in lesion changes were observed between the groups for either initial PI‐RADS 3, 4 and 5 lesions (Table 2). Furthermore, when combining PI‐RADS 4 and 5 lesions into one group, no significant difference (*p* = 0.13) in lesion change was observed between the bpMRI biopsy group and the control group: In the bpMRI biopsy group, 62% of PI‐RADS 4/5 lesions remained stable, compared to 75% in the control group. Perhaps more importantly, 85% remained as PI‐RADS 3–5 in the bpMRI biopsy group, while 79% remained as PI‐RADS 3–5 in the mpMRI biopsy group, suggesting no loss of targetable information regardless of imaging modality. In addition, bpMRI was statistically comparable to mpMRI for retention of PI‐RADS 3–5 targets within an arbitrary noninferiority margin of 10% (*p* = 0.049).

**TABLE 2:** Imaging outcomes: evolution of PI‐RADS 3–5 lesions from first to second MRI in the bpMRI biopsy group (study group) and the mpMRI biopsy group (control group).

Imaging outcomes analysis					
Changes	bpMRI biopsy group Lesions = 48		mpMRI biopsy group Lesions = 46		*p*‐value
	Number	Percentage	Number	Percentage	
Initial PI‐RADS 3 lesions	22	46%	18	39%	0.28
Stable	12	55%	12	67%	
Downgrade	3	14%	4	22%	
Upgrade	7	32%	2	11%	
Initial PI‐RADS 4 lesions	22	46%	23	50%	0.34
Stable	12	55%	17	74%	
Downgrade	8	36%	4	17%	
Upgrade	2	9%	2	9%	
Initial PI‐RADS 5 lesions	4	8%	5	11%	0.53
Stable	2	50%	4	80%	
Downgrade	2	50%	1	20%	
Initial PI‐RADS 4 or 5 lesions	26	54%	28	61%	0.13
Remained as PI‐RADS 4 or 5	16	62%	21	75%	
Downgraded to PI‐RADS 3	6	23%	1	4%	
Downgraded to <PI‐RADS 3	4	15%	6	21%	
Initial PI‐RADS 3–5 lesions	48	100%	46	100%	0.93
Remain as PI‐RADS 3–5	41	87.5%	38	82.6%	

In the biopsy outcomes analysis, we compared the bpMRI biopsy group with the matched mpMRI biopsy (control) group (Table 3). The GR rate at biopsy after the second MRI was 29% in the bpMRI biopsy group and 25% in the mpMRI biopsy group (*p* = 0.7). Extreme GR occurred in 7.3% of patients in both groups (p = 1.0). Among patients with initial GG1 disease, four in the bpMRI group and three in the mpMRI group progressed to GG3+, while among those with initial GG2 disease, one patient in the bpMRI group and two in the mpMRI group experienced extreme GR. The initial grade group distribution showed a higher proportion of GG2 cases in the bpMRI group (9 vs. 2), though this difference did not reach statistical significance (*p* = 0.06).

**TABLE 3:** Group characteristics and biopsy outcomes: rates of grade group reclassification (GR) in bpMRI biopsy group and mpMRI biopsy (control) group.

Biopsy outcomes analysis			
	bpMRI biopsy group (*N* = 68)	mpMRI biopsy group (*N* = 68)	*p*‐value
Initial grade group distribution			
Initial GG1	59	66	0.06
Initial GG2	9	2	
Grade group reclassification (any GR)	20 (29.4%)	17 (25.0%)	0.7
Extreme grade group reclassification (GG1/GG2 to GG3+)	5 (7.3%)	5 (7.3%)	1.0
GG1 → GG3+	4	3	
GG2 → GG3+	1	2	
Per lesion analysis			
PI‐RADS 3–5 lesions on second MRI	72	61	0.83
PI‐RADS 3–5 lesions biopsied	67	56	
GR per lesion (GG1 → GG2+ or GG2 → GG3+)	16 (23.8%)	12 (21.4%)	

After exclusion of lesions without documented targeted biopsy, 67 lesions in the study group and 56 in the control group were included in the per lesion analysis. GR occurred in 23.8% of lesions in the study group and 21.4% of lesions in the control group, with no statistically significant difference between groups (Table 3).

## 4 DISCUSSION

The aim of our study was to determine whether bpMRI provides comparable results to mpMRI in monitoring patients on AS, ensuring no clinically significant lesions are missed when substituting bpMRI into the AS protocol. We evaluated lesion characteristics and biopsy outcomes in patients whose first MRI was multiparametric and second was biparametric. Through both radiologic lesion and biopsy outcome analyses in matched cohorts, we assessed whether bpMRI was noninferior to mpMRI in detecting and tracking PI‐RADS 3–5 lesions.

Our findings suggest that bpMRI is a viable follow up tool in AS, yielding comparable imaging and biopsy results without loss of critical diagnostic information when all PI‐RADS 3–5 lesions are targeted during surveillance biopsies. Just as importantly, there was no significant difference in GR or extreme GR between the study group and the control group in both the overall biopsy analyses and in the per lesion analyses (Table 3). Since the primary goal of active surveillance is to identify patients with disease progression, we believe that demonstrating similar rates of GR in thoroughly biopsied AS patients followed with bpMRI compared to matched AS patients followed with mpMRI provides a compelling argument for the substitution of bpMRI once a patient is stably on AS.

In terms of radiographic findings, we compared changes in PI‐RADS 3–5 lesions on MRIs by imaging modality. In the bpMRI imaging group, there was a significant increase in number of lesions per patient at the second MRI, which makes sense given the more than two‐year median interval of time between the MRIs in men on AS (Table 1). However, the distribution of PI‐RADS scores from mpMRI to follow up bpMRI was similar Furthermore, there was no significant difference in lesion stability between the control group (mpMRI biopsy) and the experimental group (bpMRI biopsy) for patients with PI‐RADS 3–5 lesions by proportions or by noninferiority testing, suggesting that using bpMRI for follow‐up does not result in the loss of PI‐RADS 3–5 lesions.

Recent studies have highlighted the growing body of evidence supporting the use of bpMRI in the biopsy naïve setting, demonstrating its safety and diagnostic utility.^12^, ^15^, ^16^, ^17^ Several radiologic studies suggest that bpMRI can provide reliable imaging without compromising diagnostic accuracy, particularly in men without a prior prostate cancer diagnosis.^18^, ^19^, ^20^, ^21^ Considering this, it appears reasonable to extend the use of bpMRI to the follow up of patients with an existing prostate cancer diagnosis, especially when there is prior knowledge of the cancer's location based on previous biopsies or multiparametric MRI results.

**FIGURE 2:**
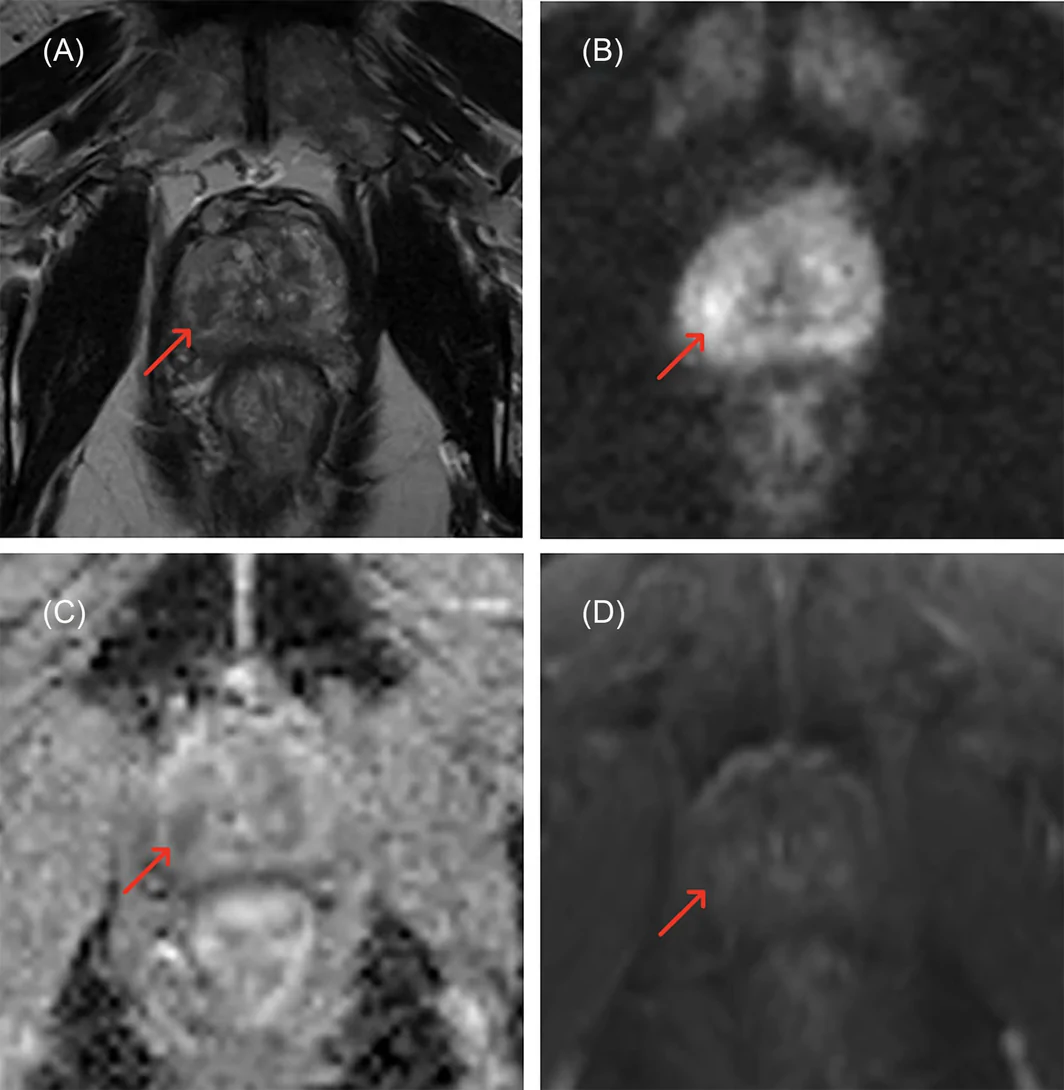
Multiparametric MRI of a PI‐RADS 4 lesion in the right apex peripheral zone: On the axial T2‐weighted image (A), the lesion shows mildly hypointense signal, and on the DWI phase (B), the lesion is moderately hyperintense. The corresponding ADC map (C) shows mild hypointensity, consistent with a PI‐RADS 3 assessment. However, the lesion demonstrates early focal enhancement on the DCE phase (D), meeting criteria for a positive DCE. According to PI‐RADS v2.1, the lesion is therefore upgraded to PI‐RADS 4.

In the context of prostate cancer, the dominant sequence for evaluating the transitional zone is T2 weighted imaging, while DWI plays a crucial role in assessing the peripheral zone. These sequences are integral components of PI‐RADS scoring, which is widely used for standardized assessment of prostate MRI.^22^ While the DCE sequence can elevate a PI‐RADS 3 lesion to a PI‐RADS 4 in the peripheral zone based on observed enhancement patterns, its utility in patients with a confirmed diagnosis of low or favourable risk prostate cancer may be less critical (Figure 2). In these cases, a bpMRI may suffice to monitor lesion progression over time, particularly when systematic and fusion prostate biopsies are also used at varying intervals during AS. The DCE being an important phase to upgrade peripheral zone PI‐RADS 3 lesions to 4 could explain why we found more downgrading of lesions 4 and 5 (as a group) to PI‐RADS 3 lesions in the bpMRI biopsy group versus the control group (Table 2). However, in AS practices that routinely biopsy PI‐RADS 3 lesions, the need for DCE imaging diminishes. In these patients, who undergo repeated biopsies as part of AS protocols, the absence of significant enhancement in the DCE sequence is less likely to alter clinical management. Thus, the use of the DCE sequence in such cases may no longer be necessary. Instead, the DCE sequence is more of a tool for monitoring patients who underwent treatment (ablation, radiation), as post‐treatment enhancement of ablated or radiated prostatic tissue can signal the presence of residual or recurrent disease.^23^, ^24^

As in the cancer‐naïve setting, our study supports the integration of bpMRI into the follow‐up of patients in AS. As the evidence base continues to grow, the use of bpMRI may expand, as will its indications and limitations, hopefully offering a more efficient, cost‐effective and patient‐friendly approach to prostate cancer monitoring that appears not significantly inferior.

Our study was unable to provide recommendations regarding long‐term oncological outcomes such as metastasis‐free survival or pathologic findings after radical prostatectomy due to the nature of the cohort and slow progression of prostate cancer. As such, we focused on radiologic changes and grade group reclassification differences between groups. In this analysis, we did not use PRECISE score and focused on PI‐RADS scores—as we have found that in low‐risk AS patients with limited follow‐up as in the present study, a patient's highest PI‐RADS score on interval MRI, rather than their PRECISE score, was a better predictor of GR on surveillance biopsy.^25^

The granularity, size and long‐standing prospective nature of our institutional AS database made this retrospective study possible. However, our study was limited by its retrospective design, potential unmeasured confounding, selection bias and a relatively short follow‐up period. Although there was heterogeneity among the radiologists, urologists and pathologists involved, this reflects real‐world clinical practice in a high‐volume prostate cancer centre. While this variability could introduce confounding, restricting the study to a single radiologist, surgeon and pathologist would have limited both cohort size and generalizability. We could not rule out selection bias in terms of which AS patients were selected for bpMRI (rather than mpMRI) for their next surveillance MRI, but we attempted to mitigate this by matching, using known predictive factors, in the creation of our control group. If anything, we thought a bias might result in lower risk patients being selected for bpMRI, but we did not find this to be the case, with a greater proportion of men with GG2 disease in the bpMRI biopsy group (though *p* = 0.06). Despite this, the rates of both overall and extreme grade reclassification were nearly identical between groups, suggesting that this modest imbalance is unlikely to have materially influenced our comparisons.

## 5 CONCLUSIONS

Incorporating bpMRI into the follow‐up of men stably on AS is comparable to using mpMRI at a tertiary referral expert centre. Matched comparison biopsy groups experienced similar rates of GR and of PI‐RADS lesion classification distribution and change. Our findings support the integration of bpMRI into AS protocols as long as all PI‐RADS 3–5 lesions are targeted at surveillance biopsy, although we cannot extend our findings to endorse bpMRI as the sole MRI modality for AS since all our patients had at least one prior mpMRI. Incorporating bpMRI into AS where possible should lead to faster studies, avoid recurrent exposure to IV contrast and increase accessibility, cost‐effectiveness and use of prostate MRI.

## AUTHOR CONTRIBUTIONS

Carlos A. Rivera López, Michelle I. Higgins, Mark N. Alshak and Joseph G. Cheaib performed the research. Patricia Landis was responsible for data entry. Katarzyna J. Macura for data quality. Yuezhou Jing, Mufaddal Mamawala and Carlos A. Rivera López analysed the data. Carlos A. Rivera López, Mohammad E. Allaf, Katarzyna J. Macura and Christian P. Pavlovich designed the study. Carlos A. Rivera López and Christian P. Pavlovich wrote the paper. Carlos A. Rivera López, Yuezhou Jing, Katarzyna J. Macura and Christian P. Pavlovich provided the final critical revisions.

## CONFLICT OF INTEREST STATEMENT

The authors declare that they have no conflicts of interest.

## Data Availability

The data that support the findings of this study are available on request from the corresponding author. The data are not publicly available due to privacy or ethical restrictions.
